# Constitution and By-Laws of the American Medical Association

**Published:** 1856-01

**Authors:** 


*Constitution and By-Laws of the American Medical Association*, in Paris France.

There is an organization of the kind in Paris, for the purpose of affording facilities to those who may hereafter go there for instruction, and also for the purpose of collecting and safe keeping American Medical Literature. In this way the French may see how much is due on this continent for medical science, of which they appear, as yet, very ignorant. We hope it may continue, for the benefit of those who visit that country, and also that it may be constituted a point for the radiation of truth and facts with regard to the progress of science in this country. A copy of its Constitution and By-Laws lies before us.

